# A novel mechanistic approach for the anti-fibrotic potential of rupatadine in rat liver via amendment of PAF/NF-ĸB p65/TGF-β1 and hedgehog/HIF-1α/VEGF trajectories

**DOI:** 10.1007/s10787-023-01147-7

**Published:** 2023-02-22

**Authors:** Manar A. Didamoony, Ahmed M. Atwa, Lamiaa A. Ahmed

**Affiliations:** 1 Pharmacology and Toxicology Department, Faculty of Pharmacy, Egyptian Russian University, Cairo, 11829 Egypt; 2 Pharmacology and Toxicology Department, Faculty of Pharmacy, Cairo University, Cairo, 11562 Egypt

**Keywords:** Diethylnitrosamine, Hedgehog, Hepatic fibrosis, HIF-1α/VEGF, PAF/NF-ĸB p65/TGF-β1, Rupatadine

## Abstract

Hepatic fibrosis is one of the major worldwide health concerns which requires tremendous research due to the limited outcomes of the current therapies. The present study was designed to assess, for the first time, the potential therapeutic effect of rupatadine (RUP) in diethylnitrosamine (DEN)-induced liver fibrosis and to explore its possible mechanistic actions. For the induction of hepatic fibrosis, rats were treated with DEN (100 mg/kg, i.p.) once weekly for 6 consecutive weeks, and on the 6th week, RUP (4 mg/kg/day, p.o.) was administered for 4 weeks. Treatment with RUP ameliorated changes in body weights, liver indices, liver function enzymes, and histopathological alterations induced by DEN. Besides, RUP amended oxidative stress, which led to the inhibition of PAF/NF-κB p65-induced inflammation, and, subsequently, prevention of TGF-β1 elevation and HSCs activation as indicated by reduced α-SMA expression and collagen deposition. Moreover, RUP exerted significant anti-fibrotic and anti-angiogenic effects by suppressing Hh and HIF-1α/VEGF signaling pathways. Our results highlight, for the first time, a promising anti-fibrotic potential of RUP in rat liver. The molecular mechanisms underlying this effect involve the attenuation of PAF/NF-κB p65/TGF-β1 and Hh pathways and, subsequently, the pathological angiogenesis (HIF-1α/VEGF).

**Graphical abstract:**

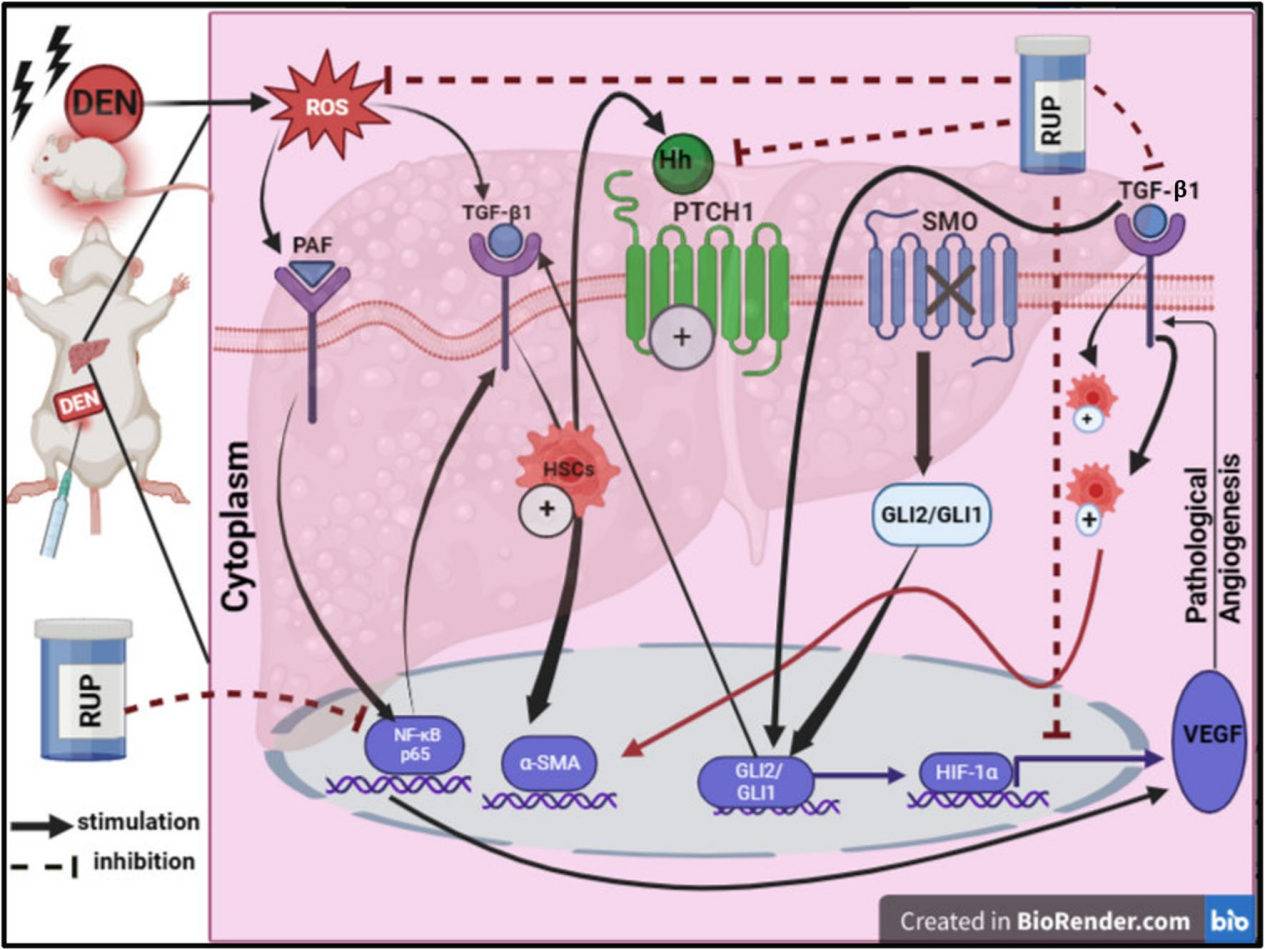

## Introduction

Liver fibrosis is a serious worldwide health problem that may progress to cirrhosis, hepatocellular carcinoma (HCC), and death. It is commonly caused by various factors like viral infections, malnutrition, alcohol abuse, biliary atresia, and hepatotoxins (Weber et al. 2016). Following chronic damage, hepatic stellate cells (HSCs) become stimulated and transdifferentiated into myofibroblast-like cells, secreting large amounts of extracellular matrix (ECM), which is linked to alpha-smooth muscle actin (α-SMA) and collagen production (Zhao et al. 2014; Cheng et al. 2018). This process impairs the material exchange between hepatocyte and hepatic sinusoidal blood (Wu et al. 2016).

Among the potent environmental hepatotoxins which induce liver fibrosis is diethylnitrosamine (DEN) (Jose et al. 1998). Liao et al. (2001) reported that DEN is found in numerous kinds of food, such as cheese, soybean, salted fish, and cured meat, in addition to alcoholic beverages. Experimentally, this model closely resembles human liver fibrosis in cellular alterations and histologic patterns (Jin et al. 2010). During its metabolic biotransformation, DEN generates reactive oxygen species (ROS), resulting in the activation of platelet-activating factor (PAF) and cellular injury (Travers 1999).

PAF is a potent lipid mediator of the inflammatory response and allergy (Borthakur et al. 2010) through binding to its G-protein-coupled receptor in hepatocytes (Karantonis et al. 2010), increasing the secretion of a variety of cytokines (Sugano et al. 2001), and leading to progression of hepatic inflammation that proceeds to fibrosis (Farombi et al. 2009). Growing evidence shows that nuclear factor-kappa B (NF-κB) stimulates fibrogenesis by releasing several growth factors like transforming growth factor-beta 1 (TGF-β1), the most potent fibrogenic stimulant for HSCs, which induces epithelial–mesenchymal transition and collagen deposition (Friedman 1999; Inagaki and Okazaki 2007).

Zhang et al. (2017) unveiled that hedgehog (Hh) signaling has been involved in the pathologic mechanisms of hepatic fibrosis. Aberrant activation of Hh signaling is reported in hepatic regeneration, vascular remodeling, fibrosis, and HCC (Omenetti et al. 2011; Zhang et al. 2017). Hypoxia is one of the main driving forces of angiogenesis where hypoxia-inducing factor-1alpha (HIF-1α), the downstream molecule of the Hh pathway, upregulates the expression of vascular endothelial growth factor (VEGF) (Feng et al. 2019). This factor plays an essential role in angiogenesis, HSCs activation, and, finally, fibrosis (Cai et al. 2021). Thus, the prevention of pathological angiogenesis can successfully inhibit the progression of liver fibrosis (Kantari-Mimoun et al. 2015).

Although studies on the pathophysiology of liver fibrosis have revealed significant progress, managing liver fibrosis is still a major health issue (Zhao et al. 2017), which requires continuous searches for new and effective therapeutic strategies (Tsochatzis et al. 2014). Rupatadine (RUP) is an approved second-generation antihistamine drug that demonstrates anti-inflammatory properties in addition to its ability to block both PAF and histaminic receptors (González-Núñez et al. 2016). Few studies have revealed its anti-fibrotic effect, where RUP ameliorated silica-induced pulmonary fibrosis (Lv et al. 2013) and isoprenaline-induced myocardial fibrosis experimentally (Ahmed et al. 2021). Most importantly, a recent study demonstrated its hepatoprotective effect against acute toxicity induced by 5-fluorouracil (5-FU) (Khalaf et al. 2022). Moreover, RUP inhibited ulcerative colitis in rats through modulation of PAF/IL-6/VEGF signaling (Ibrahim et al. 2022). Therefore, the goal of the present study was to assess the role of RUP in DEN-induced liver fibrosis and its actions on PAF/NF-κB p65/TGF-β1 and Hh/HIF-1α/VEGF signaling pathways.

## Materials and methods

### Animals

Eighteen adult male Wistar rats (8–10 weeks old), weighing 200–230 g, were purchased from the animal house of the National Research Center, Cairo, Egypt. The rats were left for one week to be accustomed at the animal house of the Faculty of Pharmacy, Cairo University, Egypt. The animals had unrestricted access to a standard diet and water throughout the experimental period. They were subjected to a controlled environment with a 12-h light/dark cycle, 23 ± 2 °C temperature, and relative humidity (60% ± 10%). The study was accepted by the Cairo University Faculty of Pharmacy Ethics Committee for Animal Experimentation [Permit Number: PT (3012)], which complies with the Guide for Care and Use of Laboratory Animals published by the US National Institutes of Health (NIH Publication, No. 85-23, revised 2011).

### Chemicals and drugs

DEN was bought from Sigma-Aldrich (St. Louis, MO, USA) and diluted in normal saline, then administered to rats intraperitoneally (i.p.) using a 23-gauge needle. RUP was obtained from Mash Premiere (Badr, Egypt), dissolved in saline, and orally (p.o.) administered using a 16-gauge round-tipped gavage p.o. needle. Unless otherwise noted, all additional chemicals were obtained from Sigma-Aldrich (St. Louis, MO, USA).

### Experimental design

Rats were randomly distributed into 3 groups (6 rats/group). The 1st group served as the normal group, which received saline intraperitoneally (once/week) for 6 weeks, followed by administration daily of oral saline for 4 successive weeks. Rats in the 2nd group received DEN (100 mg/kg, i.p) (MadanKumar et al. 2014; Essam et al. 2019) at a dose volume of 2.5 ml/kg (once/week) for 6 weeks, followed by oral administration of saline daily for 4 weeks. The 3rd group received DEN in the same regimen as group 2, followed by RUP (4 mg/kg/day, p.o.) (Lv et al. 2013; Ahmed et al. 2021), starting from the 6th to the 10th week. All animals were weighed at the start and end of the experiment. By the end of treatment, all rats were anesthetized using thiopental (50 mg/kg, i.p.). Blood samples were then obtained through the retro-orbital plexus using a sterile, non-heparinized capillary tube to separate the serum. Meanwhile, rats were sacrificed under anesthesia by cervical dislocation. Part of the liver tissues was homogenized, divided into aliquots, and frozen at − 80 °C to determine the biochemical parameters. The remaining parts were separated and sent off for histology and quantitative reverse transcription polymerase chain reaction (qRT-PCR) analyses.

### Histopathological investigation

#### Determination of the degree and area of hepatic fibrosis

The liver sections (5 µm) were stained with hematoxylin and eosin (H&E) for investigation of the morphological fibrogenic scores in accordance with the Ishak scoring system after being fixed in 10% formalin and embedded in paraffin wax (Ishak et al. 1995)**.** Additionally, sections were stained with Masson’s trichrome (Sigma-Aldrich, St. Louis, MO, USA) to reveal collagen fibers in hepatic tissues using image analysis software (ImageJ; Maryland, USA). A pathologist performed all histopathological changes in a blinded manner.

#### Immunohistochemical staining of NF-ĸB (p65) and α-SMA

Deparaffinized liver sections (5 μm) were serially dehydrated in ethanol, then treated with 5% hydrogen peroxide for 10 min. to deactivate the endogenous peroxidase. The slides were blocked for 2 h with 5% BSA in tris-buffered saline (TBS). The sections were immunostained with one of the subsequent primary antibodies: rabbit polyclonal anti-rat NF-κB p65 (Thermo Scientific, USA; Cat. #RB-9034-R7) or rabbit α-SMA polyclonal antibody to rat (ElabScience Biotechnology Inc, USA; Cat. #E-AB-34268) at a concentration of 1 μg/mL and incubated overnight at 4 °C. After rinsing slides with TBS, the sections were incubated in a solution of 0.02% diaminobenzidine containing 0.01% H_2_O_2_ for 10 min. The slides were examined using a light microscope (Olympus microscope BX-53, Crop, Tokyo, Japan) after counterstaining with hematoxylin, where the wall of blood vessels serves as an internal positive control for α-SMA (Bridle et al. 2009). Immunohistochemical quantification was carried out by assessing the percentage of the immunopositive area as indicated by the intense brown staining using image analysis software (ImageJ; Maryland, USA).

### Biochemical assessments

#### Tests of liver function

Commercial kits (Biodiagnostics, Giza, Egypt) were utilized to measure the activities of aspartate aminotransferase (AST) and alanine aminotransferase (ALT) in the separated serum in accordance with the manufacturer’s instructions.

#### Enzyme-linked immunosorbent assay (ELISA) technique for MDA, SOD, PAF, TGF-β1, HIF-1α, and VEGF evaluation

The corresponding ELISA kits were used to assess MDA (LifeSpan Biosciences; WA; USA; Cat.# LS-F28018), SOD (MyBioSource; CA, USA; Cat.# MBS036924), PAF (MyBioSource; CA, USA; Cat.# MBS2024376), TGF-β1 (MyBioSource; CA, USA; Cat.# MBS011634), HIF-1α (Kamia Biomedical; Seattle, USA; Cat.# KT-17920) and VEGF (MyBioSource; CA, USA; Cat.# MBS8506132). All steps were carried out in accordance with the manufacturers’ instructions. For the determination of protein content, the method of Lowry et al. (1951) was performed.

#### Quantitative RT-PCR analysis of Hh molecules [patched-1 receptor (Ptch1), glioma-associated oncogene homolog2 (Gli2) and glioma-associated oncogene homolog1 (Gli1)]

In brief, SV total RNA isolation system (Promega, USA) was used to extract total RNA for Hh markers. The Reverse Transcription System (Promega, USA) was then used to reverse transcribe the RNA into complementary DNA in accordance with the manufacturer’s instructions. Next, qRT-PCR was performed using SYBR Green JumpStart Taq ReadyMix (Sigma-Aldrich, USA). The primer sequences utilized for the assay are listed in Table 1. Following qRT-PCR, the 2^−∆∆CT^ formula was used to determine the relative expression of the target genes (Livak and Schmittgen 2001), where β-actin served as the housekeeping gene.

**Table 1:** The primer sequence of the target Ptch1, Gli2, and Gli1 genes

Gene	Primer sequence
Ptch1	Forward 5′-TCACAGAGACAGGGTACATGG-3′ Reverse 5′-CCCGGACTGTAGCTTTGC-3′
Gli2	Forward 5′-ATCCCCGCTTGGACTGAC-3′ Reverse 5′-ACCTCGGCCTCCTGCTTA-3′
Gli1	Forward 5′-CAGGGAAGAGAGCAGACTGA-3′ Reverse 5′-CAGGAGGATTGTGCTCCA-3′
β-actin	Forward 5′-AAGATCCTGACCGAGCGTGG-3′ Reverse 5′-CAGCACTGTGTTGGCATAGAGG-3′

### Statistical analysis

The mean ± standard error mean (SEM) was used to express the results. One-way analysis of variance (ANOVA) was then used, followed by Tukey’s post hoc multiple comparison test to interpret the data. For the histopathological scores’ evaluation, the nonparametric Kruskal–Wallis test (one-way ANOVA) and Dunn’s multiple comparison test were used to examine the results, which are expressed as median (min–max). All statistical tests and sketching of the graphs were carried out using the GraphPad Prism^®^ software package, ver. 8 (GraphPad Software, Inc., USA). The level of significance for each statistical test was performed at *p* ˂ 0.05.

## Results

### RUP treatment amended DEN-induced changes in liver indices and liver function tests in rats

Administration of DEN led to a significant reduction in the final body weight and a significant increase in liver indices (liver weight/body weight %) compared to the normal rats. Co-treatment with RUP normalized body weight as well as liver indices (Table 2). Regarding the liver enzymes, serum ALT and AST activities were significantly elevated in the DEN group by approximately fourfold and threefold, respectively, with respect to the normal group. Conversely, rats treated with RUP unveiled a significant diminish in the serum ALT and AST activities by 57% and 55%, respectively, compared to the DEN group despite being still significantly higher than the normal values (Table 2).

**Table 2:** Effect of RUP treatment on changes in body weights, liver indices, and liver enzymes in DEN-intoxicated rats

Groups	Final body wt	Liver index (%)	ALT (U/L)	AST (U/L)
Normal	281 ± 27.61	3.26 ± 0.14	26.67 ± 0.99	43.33 ± 2.03
DEN	182.7 ± 11.57*	4.36 ± 0.16*	130.7 ± 4.99*	168.3 ± 4.78*
RUP	255.7 ± 14.35^@^	3.42 ± 0.08^@^	56.33 ± 1.77*^@^	76.00 ± 2.71*^@^

Values are represented as mean (*n* = 6) ± SEM. Statistical analysis was done using one-way ANOVA followed by Tukey’s post hoc test. As compared with normal (*) and DEN (^@^) at *p* < 0.05

*ALT* alanine aminotransferase, *AST* aspartate aminotransferase, *DEN* diethylnitrosamine, *RUP* rupatadine

### RUP treatment curbed DEN-induced deterioration in the hepatic histology

**Fig. 1:**
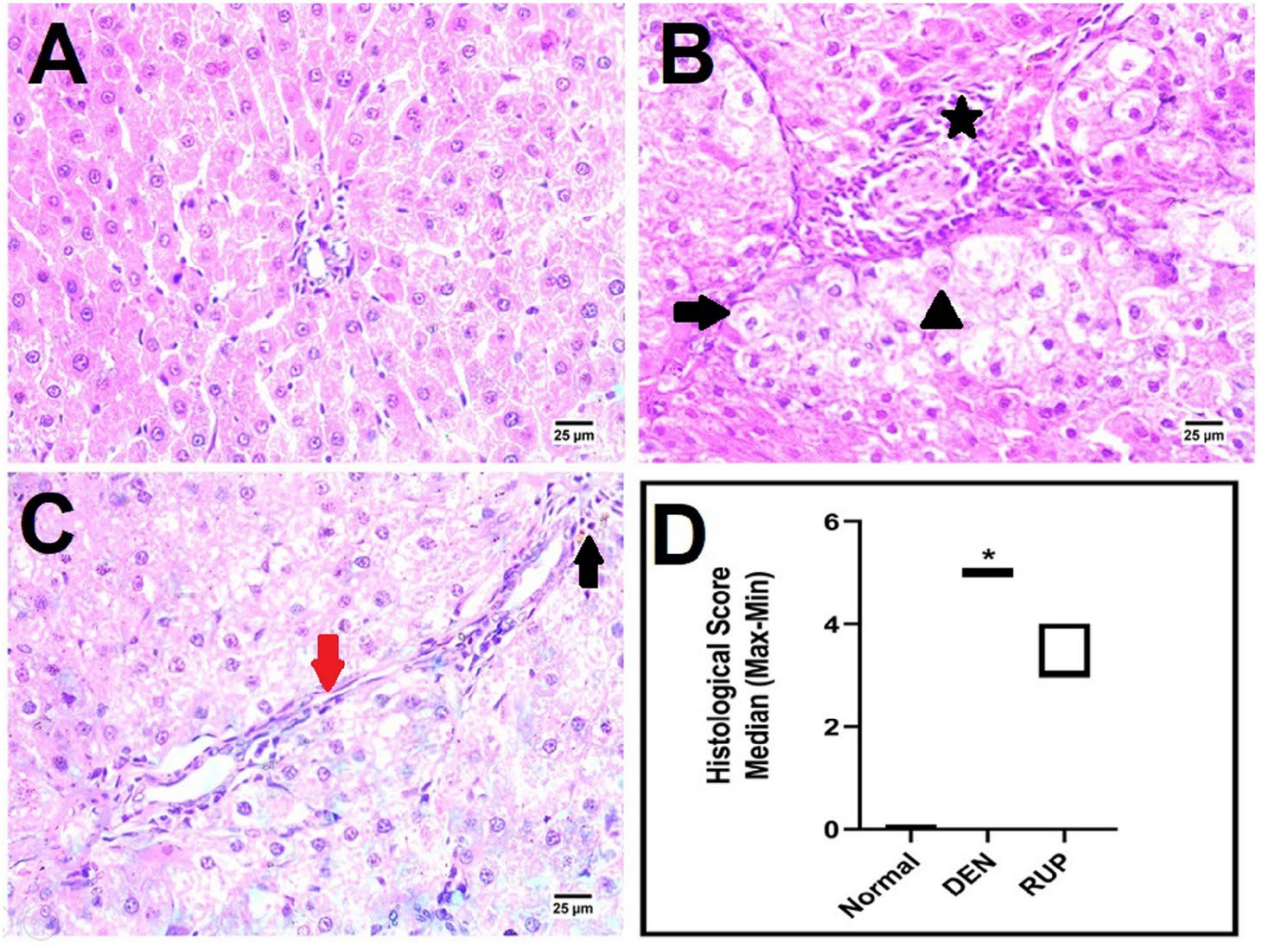
RUP treatment improved the histopathological deterioration induced by DEN. **A–D** Histopathological analysis of rat liver sections using H&E staining (× 400). **A** Normal group exhibited normal histological structure liver. **B** DEN group revealed portal infiltration with mononuclear inflammatory cells (star), vacuolar degeneration in hepatocytes (arrowhead), and formation of long connective tissue septa led to nodular formation (arrow). **C** RUP group showed portal infiltration with low numbers of mononuclear inflammatory cells (black arrow) and the formation of short connective tissue septa (red arrow). These changes are scored in panel **D**, where each value represents the median of 4 experiments (min–max). Statistical analysis was carried out using the Kruskal–Wallis test (one-way ANOVA) followed by Dunn’s multiple comparison test, as compared to normal (*) and DEN (^@^) groups at *p* ˂ 0.05. *DEN* diethylnitrosamine, *H&E* hematoxylin and eosin, *RUP* rupatadine

According to Fig. 1A, the liver’s photomicrographs revealed the normal hepatic architecture of the central vein as well as surrounding hepatocytes in normal rats. Still, those present in the DEN group demonstrated hepatic nodules due to the formation of long fibrous connective tissue septa with infiltration of the portal area by mononuclear inflammatory cells infiltration and the presence of vacuolar degeneration in hepatocytes (Fig. 1B). On the contrary, RUP treatment revealed a marked amelioration in portal fibrosis by decreasing number of mononuclear inflammatory cells infiltration in a portal area with the disappearance of hepatic nodules and formation of short connective tissue septa (Fig. 1C).

### RUP treatment attenuated DEN-induced oxidative stress

SOD activity was reduced by 81% in the DEN group; meanwhile, a significant elevation of the hepatic lipid peroxidation product (MDA) (about sixfold) was obvious compared to the normal group. RUP-treated rats showed about a threefold elevation of SOD activity together with a significant reduction of hepatic MDA content by 58%, compared to the DEN group (Table 3).

**Table 3:** Effect of RUP treatment on oxidative stress markers in DEN-intoxicated rats

Groups	SOD (U/mg protein)	MDA (ng/mg protein)
Normal	67.20 ± 1.12	7.80 ± 0.32
DEN	14.30 ± 0.54*	43.50 ± 1.46*
RUP	41.70 ± 0.84*^@^	18.10 ± 0.45*^@^

Values are represented as mean (*n* = 6) ± SEM. Statistical analysis was done using one-way ANOVA followed by Tukey’s post hoc test. As compared with normal (*) and DEN (^@^) at *p* ˂ 0.05

*DEN* diethylnitrosamine, *MDA* malonaldehyde, *RUP* rupatadine, *SOD* superoxide dismutase

### RUP treatment ameliorated DEN-induced hepatic inflammatory events

**Fig. 2:**
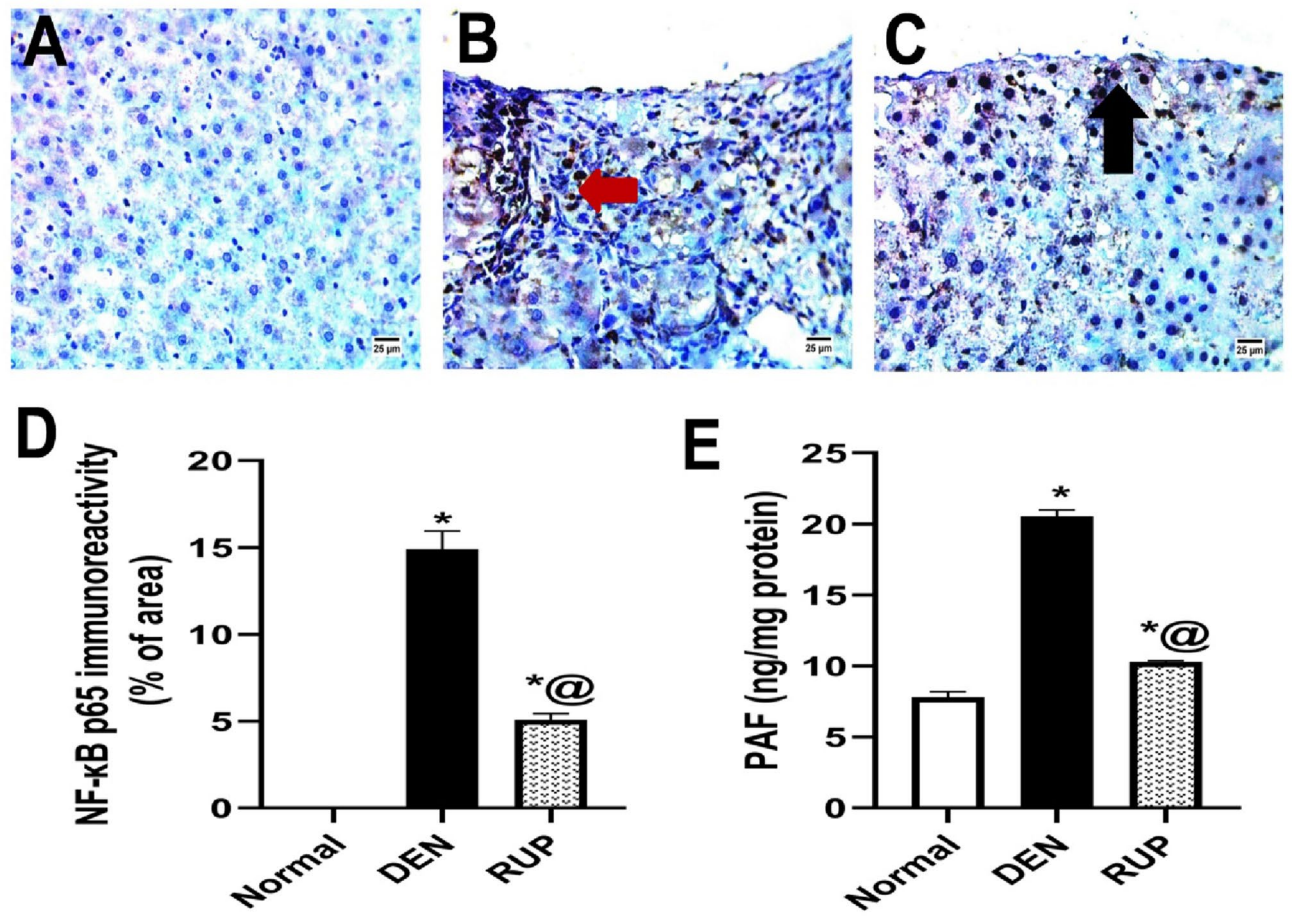
RUP treatment decreased the hepatic inflammation induced by DEN. **A**–**C** Immunohistochemical detection of NF-κB p65 (brown stain) (× 400). **A** Normal group showed no NF-κB expression. **B** DEN group revealed a significant increase in NF-κB p65 expression in nuclei and cytoplasm of hepatocytes (arrow). **C** RUP group demonstrated less NF-κB expression in nuclei of hepatocytes (arrow). **D** Quantitative image analysis of NF-κB p65 immunoreactivity (% area). **E** Effect of RUP on the hepatic PAF content as determined using a commercial ELISA kit. Values are represented as mean (*n* = 4 for histological examination and *n* = 6 for PAF estimation)  ± SEM. Statistical analysis was done using one-way ANOVA followed by Tukey’s post hoc test. As compared with normal (*) and DEN (^@^) at *p* *˂* 0.05. *DEN* diethylnitrosamine, *ELISA*: enzyme-linked immunosorbent assay, *NF-κB p65* nuclear factor-kappa B p65, *PAF* platelet-activating factor, *RUP* rupatadine

DEN exposure led to an upsurge of the inflammatory response (Fig. 2), as reflected by a marked increment of immunohistochemical NF-ĸB p65 expression (Fig. 2A–D) to 15% as well as a significant elevation of the hepatic PAF content to approximately threefold (Fig. 2E). On the other hand, co-treatment with RUP significantly attenuated these effects.

### RUP treatment mitigated DEN-induced hepatic fibrogenesis

**Fig. 3:**
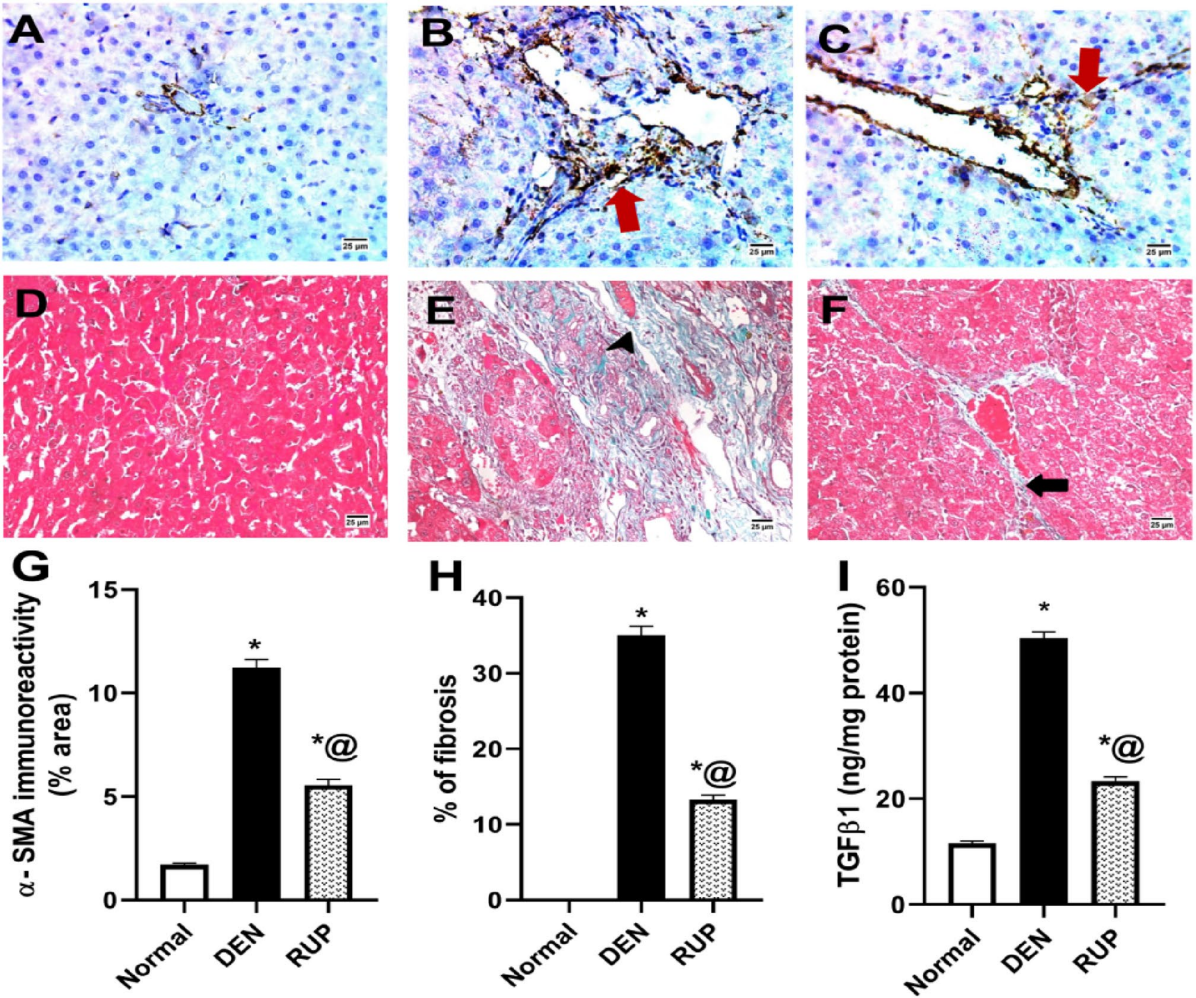
RUP treatment decreased the hepatic fibrosis induced by DEN. **A**–**C** Immunohistochemical detection of α-SMA (× 400) as an indicator for HSCs activation (brown color). **A** Normal group showed an absence of α-SMA expression in the hepatic parenchyma. **B** DEN group revealed extensive α-SMA expression in the portal area, indicating enormous numbers of activated HSCs (arrow). **C** RUP group clarified mild α-SMA expression in hepatic parenchyma (arrow). **D–F** Specimens stained with Masson’s trichrome to assess hepatic fibrosis (blue color). **D** Normal group showed a normal histological picture of the liver, **E** the DEN group showed severe portal fibrosis (arrowhead), and **F** the RUP group showed only short connective tissue septa between two portal areas (arrow). **G** Quantitative image analysis for α-SMA (% area). **H** Image analysis of % of fibrosis for Masson’s trichrome specimens. **I** Effect of RUP on the hepatic TGF-β1 content was determined using the relevant ELISA kit**.** Values are represented as mean (*n* = 4 for histological examination and *n* = 6 for TGF-β1 estimation) ± SEM. Statistical analysis was done using one-way ANOVA followed by Tukey’s post hoc test. As compared with normal (*) and DEN (^@^) at *p* *˂* 0.05. *α-SMA* alpha smooth muscle actin, *DEN* diethylnitrosamine, *ELISA* enzyme-linked immunosorbent assay, *RUP* rupatadine, *TGF-β1* transforming growth factor-beta1

According to Fig. 3, which depicts the immunohistochemical detection of α-SMA, a significant amount of brown staining (11.22% of the immunopositive area) was revealed in the DEN group. On the other hand, RUP significantly decreased the positively stained area of α-SMA. Moreover, RUP’s anti-fibrotic effect was further proved by the histological visualization of collagen fibers using Masson’s trichrome stain, where the increase in collagen deposition induced by DEN was markedly alleviated by RUP as clarified in Fig. 3D–F. Additionally, ELISA analysis revealed a higher hepatic TGF-β1 content (about fourfold) in the DEN group than in the normal group. RUP treatment demonstrated a significant decrease in TGF-β1 content by 54%, compared with the DEN group Fig. 3I.

### RUP treatment suppressed the extensive activation of Hh trajectory induced by DEN

**Fig. 4:**
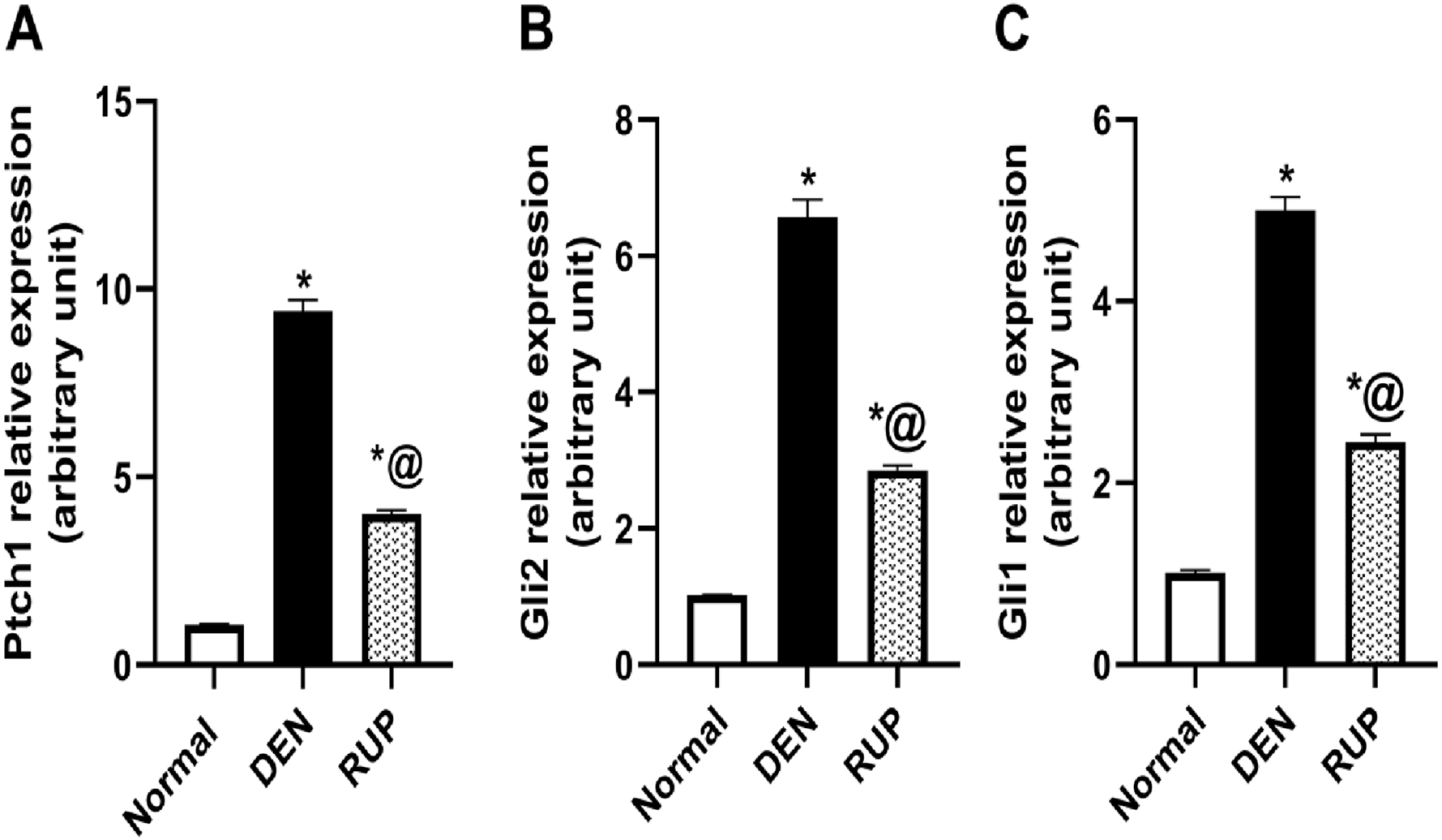
RUP treatment blocked the Hh trajectory induced by DEN administration, as revealed by its effects on mRNA expression of **A** Ptch1, **B** Gli2, and **C** Gli1. Values are represented as mean (*n* = 6) ± SEM. Statistical analysis was done using one-way ANOVA followed by Tukey’s post hoc test. As compared with normal (*) and DEN (^@^) at *p* *˂* 0.05. *DEN* diethylnitrosamine, *Gli1* glioma-associated oncogene homolog1, *Gli*2 glioma-associated oncogene homolog2, *Ptch1* patched-1 receptor, *RUP* rupatadine

As illustrated in Fig. 4, DEN turned on the Hh pathway, as revealed by the significant increase in Ptch1, Gli2, and Gli1 mRNA expressions to approximately   nine-, six-, and five-fold, respectively, compared with the normal group. Strikingly, co-treatment with RUP markedly curtailed the genetic expressions of Ptch1, Gli2, and Gli1 by 57%, 57%, and 51%, respectively, compared with the DEN group, confirming the inhibitory effect of RUP on the Hh pathway.

### RUP treatment inhibited the activation of the HIF-1α/VEGF pathway

**Fig. 5:**
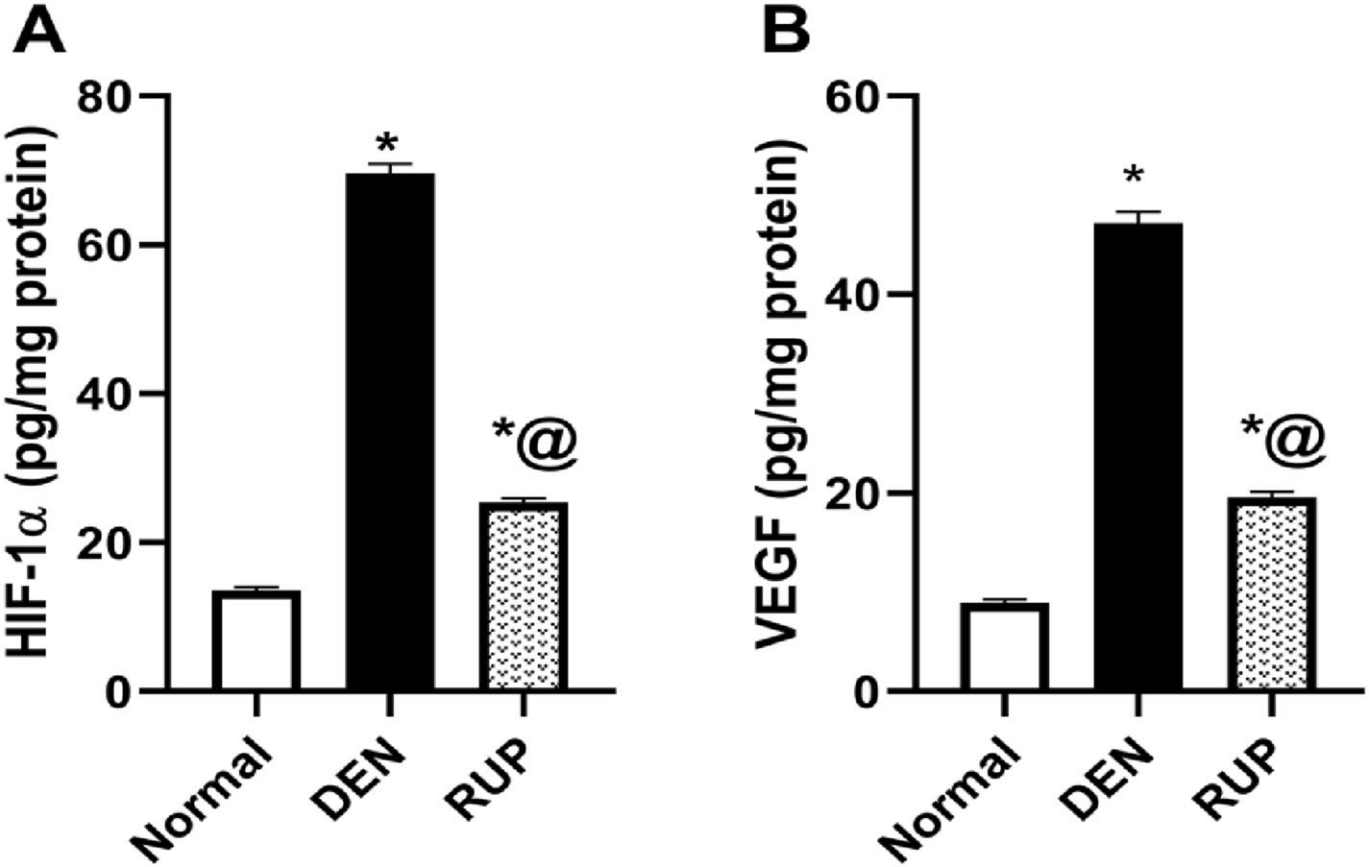
RUP treatment inhibited the activation of the HIF-1α/VEGF pathway induced by DEN administration, as revealed by its effects on hepatic contents of **A** HIF-1α and **B** VEGF. Values are represented as mean (*n* = 6) ± SEM. Statistical analysis was done using one-way ANOVA followed by Tukey’s post hoc test. As compared with normal (*) and DEN (^@^), at *p* ˂ 0.05. *DEN* diethylnitrosamine, *HIF-1α* hypoxia inducing factor-1alpha, *RUP* rupatadine, *VEGF* vascular endothelial growth factor

DEN-treated group markedly increased the hepatic contents of HIF-1α and VEGF by approximately fourfold (Fig. 5). Treatment with RUP signified its action on the pathological angiogenesis by diminishing HIF-1α and VEGF contents by 64% and 58%, respectively, compared to the DEN-treated group.

## Discussion

Hepatic fibrosis is a complex universal health problem with significant morbidity and mortality rates worldwide due to its fast development into cirrhosis and HCC (Ogaly et al. 2022). Upon hepatic fibrosis, the dormant HSCs transdifferentiate into myofibroblast-like cells with loss of vitamin A lipid droplets, augmented α-SMA expression, and overproduction of ECM components such as collagens, proteoglycans, and glycoproteins (Perumal et al. 2017).

In the current study, the liver fibrosis model was induced by DEN. This model closely mimics the human liver fibrosis’s cellular alterations and histologic features (Jin et al. 2010). DEN-induced liver fibrosis causes loss and rupture of hepatocyte membrane functional integrity, which speeds up the leakage of liver enzymes (ALT and AST) into the bloodstream (Jin et al. 2019). These markers are usually considered direct sensitive biomarkers for evaluating hepatocellular damage (Moreira et al. 2015). Similarly, the histopathological findings of DEN-treated rats provided additional evidence of liver injury and demonstrated significant deviations from the normal patterns. These changes involved severe portal fibrosis with the nodular formation and high numbers of mononuclear inflammatory cell infiltration. Notably, these findings concur with earlier research (Essam et al. 2019).

Treatment with RUP exerted notable therapeutic effects against DEN-elicited fibrosis, as evidenced by significant suppression of ALT and AST activities. Histopathological examination of RUP showed a remarkable improvement and alleviation of portal fibrosis and mononuclear inflammatory cell infiltration compared to the DEN group. In the same context, Khalaf et al. (2022) showed that the RUP provided normal liver architecture and a significant decrease in ALT and AST activities in an experimental model of 5-FU-induced acute hepatotoxicity in rats. RUP could stabilize the membrane structure of hepatocytes, thereby preventing the cellular damage induced by DEN.

In the present study, body weight loss as well as increasing in liver weight index were observed in DEN-exposed rats during the experimentation period. Noticeably, the reduction in body weight could be due to the diminished appetite and decreased food intake caused by DEN (Farazi and DePinho 2006). The increase in liver weight index could also be related to the provoked inflammation in hepatotoxin-exposed animals with an accumulation of ECM and inflammatory cell infiltration (El-Mihi et al. 2017). Rodents treated with RUP preserved their body weights and showed marked suppression in liver indices compared to the DEN group. These results support the histopathological and biochemical results observed in the RUP group.

The present findings indicate that RUP has potent anti-oxidant and free radical scavenging activities, as evidenced by a significant decrease in the hepatic MDA content and an increase in SOD activity compared to the DEN group. It is well-recognized that oxidative stress increases collagen deposition, contributing to liver fibrosis development (Yang et al. 2019). Growing evidence has demonstrated that DEN is metabolized by cytochrome P450 2E1, elevating the production of ROS (Essam et al. 2019), enhancing lipid peroxidation, causing further damage and overwhelming the anti-oxidant enzymatic (such as SOD) and non-enzymatic defense systems. All these consequences contribute to cellular damage (Motawi et al. 2011). RUP previously alleviated ROS-induced hepatocyte and intestinal apoptosis (Mohamed and Mohammed 2021). Besides, the anti-oxidant activity of RUP was demonstrated in rats with acute pancreatitis and diabetic nephropathy as well as in allergic rhinitis in patients (Hafez et al. 2020; Kahveci et al. 2021; Mohamed et al. 2022). Based on these findings, the anti-oxidant activity of RUP could be postulated to prevent ROS-induced fibrosis.

In the current investigation, DEN intoxication disrupted the redox balance in favor of increased ROS, which increases the hepatic content of PAF, as demonstrated earlier by Hwa Choi et al. (2000) and Ajiboye et al. (2013). On the other hand, RUP significantly prevented PAF activation, mainly through the inhibition of oxidative stress. PAF is an upstream regulator and a proximal stimulator of NF-κB. The activation of PAF stimulates the activation and nuclear translocation of NF-κB p65, leading to vascular smooth muscle cell proliferation (Ibe et al. 2008; Ogbozor et al. 2015). Additionally, NF-κB increases the expression of various pro-inflammatory cytokines like tumor necrosis factor-alpha (TNF-α) that induce further stimulation of NF-κB level, provoking the inflammatory process (Sasaki and Iwai 2016). Pro-inflammatory mediators also increase phospholipase A2 synthesis, transforming lysophospholipids to PAF (Pfeilschifter et al. 1993; Tran et al. 2018). Importantly, the oxidative stress ameliorated by RUP could contribute mainly to preventing this inflammatory status by inhibiting NF-κB (Köhler et al. 2016; Herpers et al. 2016; Wang et al. 2018). In the same context, Mohamed and Mohammed (2021) showed that RUP suppressed PAF content and TNF-α/NF-κB p65 pathway in 5-FU-induced intestinal toxicity of rats. Moreover, RUP notably repressed macrophage and mast cell infiltration and inhibited expression of TNF-α and mast cell degranulation in a rat model of lung fibrosis (Lv et al. 2013). Similarly, our findings showed that RUP inhibited the hepatic PAF content, suppressed NF-κB p65 expression, and prevented the subsequent inflammatory cascade, providing evidence for the anti-inflammatory effect of RUP.

Besides upsurging the inflammation process, NF-κB plays a significant role in activating HSCs (Elsharkawy and Mann 2007; El-Agroudy et al. 2016) by releasing several growth factors like TGF-β1. TGF-β1 regulates the expression of α-SMA, a reliable marker of HSCs activation (Desmouliere et al. 1993; Abdel-Rahman et al. 2019). TGF-β1 also induces fibrosis by supporting the differentiation of inactive fibroblasts into matrix-secreting myofibroblasts (Vaughan et al. 2000; Lijnen et al. 2003) and increasing the synthesis of collagen (Epstein et al. 1994; Koh et al. 2015) in the ECM, exacerbating hepatic fibrosis (George et al. 2019). In this study, the hepatic content of TGF-β1 and α-SMA immunostaining  were significantly perturbated in hepatic tissues of DEN-treated rats, which concurred with the results of earlier studies (Perumal et al. 2017; Cheng et al. 2018; Husain  et al. 2018). These data were also confirmed by Masson’s trichrome staining, which revealed a massive deposition of collagen fibers in DEN-exposed rats.

Based on its obvious anti-oxidant and anti-inflammatory effects, RUP treatment significantly decreased the hepatic content of TGF-β1, inhibiting HSCs activation as shown by reduced α-SMA expression. Following the same pattern, the inhibition of ROS was previously shown to diminish TGF-β1-spurred α-SMA expression in rat hepatic fibroblast (Yao et al. 2012; Calleja et al. 2013). NF-κB inhibition also formerly abated the elevation of TGF-β1 and its mediated HSCs activation (Mao et al. 2015). The amelioration of these inflammatory and fibrogenic markers by RUP was confirmed by evident and marked amelioration of the degree of liver fibrosis as indicated by the significant reduction of α-SMA expression and collagen deposition. These results are consistent with previous research demonstrating the anti-fibrogenic potential of RUP in models of pulmonary and cardiac fibrosis (Lv et al. 2013; Ahmed et al. 2021).

Our findings revealed for the first time that RUP is a potent Hh cascade inhibitor, as shown by the marked reduction in expressions of Ptch1, Gli2, and Gli1. Hh pathway has been implicated in the progression of liver fibrosis (Sicklick et al. 2005; Guy et al. 2012; Yang et al. 2014). In normal liver tissues, this axis is inert where Hh ligands are absent (Omenetti et al. 2011). Meanwhile, in liver fibrosis, the activated HSCs induced by TGF-β1 are capable of producing Hh ligands that bind to their patched receptor (Choi et al. 2011; Feng et al. 2019), releasing smoothened transmembrane protein (SMO) which stimulates the activation and nuclear translocation of the Gli transcription factors (Jiayuan et al. 2020). Among these factors, Gli2 induces Gli1 activation, which serves again as a signal amplifier downstream of Gli2 (Kramann 2016), triggering the signaling of a plethora of target genes involved in myofibroblast formation such as α-SMA and TGF-β1. TGF-β1 is a potent inducer of Gli2 transcription (Syn et al. 2009; Choi et al. 2010) and Hh pathway (Jiayuan et al. 2020). In the same context, the current results reveal the correlation between the hepatic fibrosis prompted by DEN and the marked stimulation of Hh signaling, as clarified by the amplified gene expressions of Ptch1 as well as Gli1 and Gli2.

RUP principally attenuated Hh signaling by inhibiting TGF-β1 content, whereas the latter inhibition is known to turn off Hh signaling (Mansour et al. 2021). The inhibition of the Hh pathway downregulates the myofibroblast-related genes and causes the reversal of myofibroblasts to a quiescent phenotype, thus attenuating fibrosis (Pratap et al. 2012). GANT61, a Gli inhibitor, consistently proved an anti-fibrotic effect against CCl4-induced hepatic fibrosis in mice (Jiayuan et al. 2020). Inhibition of the Hh hub with vismodegib, a SMO antagonist, also stimulated the regression of both hepatic fibrosis and HCC (Philips et al. 2011).

Several studies highlighted the inhibition of hepatic angiogenesis as a novel target for treating hepatic fibrosis (Zhou et al. 2014; Feng et al. 2019). In the present study, the anti-angiogenic activities of RUP in the DEN model were confirmed by significant suppression of the measured HIF-1α/VEGF signaling which was markedly elevated in the DEN group. Noteworthy, the hypoxia in the fibrotic liver could be attributed to the sinusoids’ structural changes, including basement membrane deposition and loss of liver sinusoidal endothelial cells (LSECs) fenestrae. These changes lead to impaired oxygen diffusion from the sinusoids to the parenchyma (Rosmorduc and Housset 2010), where hypoxia initiates an intracellular pathway leading to the activation of the transcription factor HIF-1α. Additionally, several factors, such as ROS and inflammatory cytokines, could activate HIF-1α accumulation (Ushio-Fukai and Alexander 2004; Bonello et al. 2007; Moczydlowska et al. 2017). Noteworthy, in liver fibrosis, Gli1 has been clarified as a promoter for HIF-1α (Zhang et al. 2017; Yang et al. 2020). HIF-1α also binds to hypoxia-responsive elements found in the promoter of several target genes, including VEGF (Forsythe et al. 1996; Bozova and Elpek 2007), where VEGF directly stimulates LSECs and provokes angiogenesis together with activation of HSCs (Yin et al. 2013). HSCs also express several CXC chemokines, which can manipulate angiogenesis (Hickey and Simon 2006), supporting the positive correlation of angiogenesis with fibrogenesis (Feng et al. 2019). Similar to our RUP results, curcumin inhibited liver fibrosis in rats by suppressing HIF-1α/VEGF-induced angiogenesis (Zhang et al. 2014). As well, Ibrahim et al. (Ibrahim et al. 2022) reported that RUP downregulated VEGF content in the ulcerative colitis model via inhibition of PAF. PAF could stimulate angiogenesis by releasing NF-ĸB- dependent angiogenic factors such as VEGF (JH et al. 2001).

## Conclusion

In summary, the present study may satisfy an unmet need in developing a therapeutic agent for the treatment of hepatic fibrosis. RUP is a safely prescribed drug that demonstrated a favorable anti-fibrotic effect in rat liver for the first time in the current study. The molecular mechanisms underlying this protective effect involve attenuating oxidative stress, inflammation, and fibrosis by inhibiting PAF/NF-κB p65/TGF-β1 axis. More importantly, RUP inhibited liver fibrosis not only through suppression of HSCs activation and ECM deposition but also through Hh pathway impediment as well as its anti-angiogenic effect via inhibition of HIF-1α/VEGF trajectory. Further experimental studies are required to confirm the anti-fibrotic potential of RUP in the liver and to elucidate other underlying mechanisms before starting any clinical trials.

## Data Availability

The data sets generated during and/or analyzed during the current study are available from the corresponding author upon reasonable request.
